# The Individual Differences in the Perception of Oral Chemesthesis Are Linked to Taste Sensitivity

**DOI:** 10.3390/foods10112730

**Published:** 2021-11-08

**Authors:** Sulo Roukka, Sari Puputti, Heikki Aisala, Ulla Hoppu, Laila Seppä, Mari A. Sandell

**Affiliations:** 1 Department of Food and Nutrition, University of Helsinki, 00014 Helsinki, Finland; sulo.roukka@helsinki.fi (S.R.); laila.seppa@helsinki.fi (L.S.); 2 Functional Foods Forum, University of Turku, 20014 Turku, Finland; sari.puputti@utu.fi (S.P.); heikki.aisala@vtt.fi (H.A.); ulla.hoppu@utu.fi (U.H.)

**Keywords:** chemesthesis, chemesthesis sensitivity, hierarchical clustering, individual differences, intensity, perception, sensitivity, taste sensitivity

## Abstract

Chemesthesis is a part of the flavor experience of foods. Chemesthetic perception is studied to understand its effect on food-related behavior and health. Thus, the objective of this research was to study individual differences in chemesthetic perception. Our study involved sensory tests of three chemesthetic modalities (astringency, pungency, and cooling). Participants (*N* = 196) evaluated the intensity of samples in different concentrations using a line scale under sensory laboratory conditions. Aluminum ammonium sulfate, capsaicin, and menthol were used as the prototypic chemesthetic compounds. The participants were divided into sensitivity groups in different chemesthetic modalities by hierarchical clustering based on their intensity ratings. In addition, an oral chemesthesis sensitivity score was determined to represent the generalized chemesthesis sensitivity. The results showed that people can perceive chemesthesis on different intensity levels. There were significantly positive correlations between (1) sensitivity scores for oral chemesthesis and taste as well as (2) each chemesthesis and taste modalities. Moreover, based on the multinomial logistic regression model, significant interactions between oral chemesthesis and taste sensitivity were discovered. Our findings showed that people can be classified into different oral chemesthesis sensitivity groups. The methods and results of this study can be utilized to investigate associations with food-related behavior and health.

## 1. Introduction

Human senses contribute directly to food choices [1,2]. Chemesthesis together with taste and smell are classified into chemical sensations. They form flavor experiences of foods that influence food choices immediately and in the long term [1]. ASTM-WK44511 (E253-21, 2014) has defined that chemesthesis is a sensory sensitivity to direct chemical stimulation of touch, pain, and thermal receptors in the skin and mucous membranes that cover all over the body including all types of skin as well as the nose, mouth, and eyes [3]. Moreover, oral chemesthetic perception arises primarily from trigeminal stimulation [4]. This creates a variety of flavor experiences such as pungency of chili peppers [4], cooling of hygiene products [5], and astringency of berries [6].

Chemesthetic compounds protect plants against mammals and insects by causing strong somatosensory stimuli that signal a threat [7]. In addition, some plants contain volatile oils and terpenes that protect against pathogenic microbes [7]. Slack (2016) states [8] that chemesthetic perception helps animals to avoid harmful substances. Humans, however, tend to use spices containing chemesthetic irritants to improve flavor experiences of foods and beverages [8].

The mucous membranes of human beings are stated to be the most sensitive area to chemesthetic stimuli due to the high number of receptors that are involved in chemesthetic perception [9]. Different modalities of chemesthesis can be stimulated in the oral cavity area. The oral chemesthetic irritants are primarily detected via three cranial nerves: *Trigeminal* (V), *Glossopharyngeal* (IX), and *Vagus* (X) [10]. Furthermore, these nerves are activated by a family of receptors (transient receptor potential channels, TRP) that normally convey senses such as temperature, pain, touch, or texture [8]. Therefore, chemesthesis differs from taste perception. Slack (2016) has argued [8] that chemesthesis is easily overlooked in considerations of taste and smell. Generally, the chemosensory literature seems to be dominated by taste and smell research [10].

Despite the profusion of work identifying mechanisms of chemesthesis, the measurements of the oral chemesthesis sensitivity are still lacking in scientific literature. However, different chemesthetic modalities and irritants have been studied and measured individually. For example, these include capsaicin of chili peppers and polyphenolic compounds of berries [6] that are commonly consumed and found in foods. Additionally, a recent study has shown that individual variations in taste modalities (sweet, sour, salty, bitter, and umami) and astringency of tannic acid seem to interact with dietary intake and preferences for fruits and vegetables [11]. Therefore, chemesthetic agents in foods can have a strong effect on our food-related behavior that includes, e.g., food consumption, acceptance, preferences, and choices. Piochi et al. (2020) highlight that oral pungency and spicy sensations affect food-related behavior and diets, and in addition, the nasal responsiveness to irritants may contribute to influencing food acceptance [12]. This may be explained by their direct stimulation of pain receptors since capsaicin and menthol can stimulate pain perception [8,13]. Thus, the utilization of different chemesthetic chemicals needs to be understood and carefully designed for sensory tests. Nevertheless, there is likely more than one factor explaining the differences in the oral chemesthesis sensitivity.

Moreover, the oral sensitivity measurements that are relevant to food, primarily focus on classifying individual sensitivity to PROP (6-n-propylthiouracil) taste sensitivity [14,15]. However, that is measured with only one bitter compound. In addition, the studies focusing on analyzing taste sensitivity (measured with a series of taste modalities: sweet, sour, salty, bitter, and umami) are quite rare still [14,16]. Puputti et al. (2018) have found out that sensitivity to bitter was linked to the semi-sensitive perception of sourness, and insensitivity to umami was a significant predictor of the bitter insensitivity [14]. Furthermore, the sour insensitivity was also a significant predictor for umami insensitivity. In addition, the sour sensitivity predicted sensitivity to sweetness and saltiness. Based on the taste sensitivity score (TSS) that takes into account five taste modalities at the same time, people may be classified into hyposensitive, semi-sensitive, and hypersensitive tasters [14]. However, these sensitivity measurements focus only on taste perception. The oral responsiveness to taste (sweetness of sucrose, sourness of citric acid, saltiness of sodium chloride, and bitterness of caffeine) and chemesthesis (pungency of capsaicin and astringency of potassium aluminum sulfate) sensations has been stated to be linked to the consumption and preferences of alcoholic beverages [17,18]. Therefore, the oral chemesthesis sensitivity needs to be examined to understand its possible role of individual differences in food-related behavior and health.

Astringency is a multidimensional perception that can be activated by a wide range of compounds found in food [19]. Therefore, the overall astringency can be divided into several subqualities. The astringency of aluminum sulfate differs from the astringency of tannic acid [19,20,21]. In addition, aluminum does not decrease the viscosity of saliva and thus does not interact with salivary proteins in the same way as tannic acid. In the case of astringency elicited by aluminum, early wine-related studies have highlighted the possibility that alum sulfate activation might be related to PROP-taster status [21]. Furthermore, later studies have proven that the astringency of aluminum compounds (such as aluminum sulfate) can be perceived more intensely by PROP-sensitive compared to non-sensitive individuals, especially when using high concentrations [19,22,23]. Moreover, Louro et al. (2021) has discovered that the intensity between tastes modalities and tannic acid elicited astringency is mostly in positive correlation, despite the lack of correlation with the perception of saltiness [11].

Pungency perception can be activated by capsaicin (8-methyl-N-vanillyl-6-nonenamide) which is a compound found naturally in chili peppers [24]. Earlier studies have indicated that bitter taste and pungency perception from capsaicin are qualitatively similar [25]. Furthermore, it is known that capsaicin can activate bitterness as a secondary stimulus in addition to pungency [26], and, hence, studies show that the variability in perceived bitterness of capsaicin was significantly associated with taste receptor TAS2R38 and TAS2R3/4/5 diplotypes [27]. The individual sensitivity level to general pungency can vary dependent on the different irritants and activation mechanisms.

Cooling perception can be activated by a variety of different compounds such as menthol (5-Methyl-2-(propan-2-yl)cyclohexan-1-ol), which can be found naturally in mint [28]. In addition, menthol is known and commonly used in food and hygiene products such as chewing gum, toothpaste, and mouthwash due to its cooling properties. It has been studied that menthol and capsaicin can stimulate a subset of taste neurons, which respond to bitter substances [29]. It has been shown that menthol and sweet taste increase cough reflex, which could indicate an interaction between sweet taste and menthol [30].

This research is a part of the FoodTaste project, which focuses on studying individual differences among human beings in sensory perception. The objective of this study was to explore individual differences in chemesthetic perception and test whether the oral chemesthesis sensitivity can be measured. The hypotheses were that (1) the oral chemesthesis sensitivity correlates with taste sensitivity [14], which was measured earlier in the same project by Puputti et al. (2018), and (2) people can be classified into different chemesthesis sensitivity groups. The methodology included rating the intensity of different liquid chemesthetic samples on a line scale and analyzing the collected data with hierarchical clustering which classified participants into chemesthesis-specific sensitivity groups.

## 2. Materials and Methods

### 2.1. Participants

The sensory evaluation test was conducted at the University of Turku, Functional Foods Forum sensory laboratory (ISO 8589). The recruitment was public, and the exclusion criteria were allergies, pregnancy, and a lactating state. As a result, 205 voluntary adults participated in the chemesthesis sensory evaluation test.

The participants were instructed not to eat, drink anything other than water, chew gum, nor smoke for one hour before the session. Before the sensory evaluation, they signed a consent form that included information about the structure and objectives of the study. The subjects were untrained and received written and oral instruction before every section of the tests.

The sensitivity measurement of oral chemesthesis was studied from the same participants that participated in the taste sensitivity study [14] every couple of weeks. Moreover, we used similar sensory evaluation and data processing methods to achieve a valid and reliable comparison with taste sensitivity.

After the sensory evaluation visit, participants received a reward. Only coded ID numbers were used in the data analysis steps to secure participants’ personal information and identity. The study was reviewed by the Southwest Finland Hospital District’s Ethics Committee (145/1801/2014) and follows the European Union’s General Data Protection Regulation (GDPR).

### 2.2. Sample Preparation

The chosen chemesthetic modalities were astringency, pungency, and cooling, which were tested and analyzed for this research. One prototypic compound on each modality was chosen. Thus, three different sample series were created with a total of six samples per modality, including five concentration levels determined experimentally by the previous experiences using quarter logarithmic dilution series (Table 1) and one water sample as a zero-sample.

The samples were diluted by using active carbon filtered water and stored under refrigeration in glass bottles with good laboratory practice. Since capsaicin and menthol are hydrophobic compounds, they were first diluted into glyceryl tri-acetate solution. After that, the created stock solutions were diluted with active carbon filtered water into the samples. Concentration levels of glyceryl triacetate were pretested to avoid smell and taste in the sensory samples. All the solutions were prepared less than four days before the evaluation session. Every sample was settled into room temperature before serving.

### 2.3. Sensory Evaluation Procedure

The sensory evaluation data was collected by using Compusense five Plus 5.6 software (Compusense, Guelph, ON, Canada) in the sensory laboratory and background information (gender and age) with Webropol (Webropol Inc., Helsinki, Finland) online questionnaires.

Before the sensory evaluation, the participants were informed of the tested chemesthetic stimuli by written and oral information. They were instructed to the sensory evaluation procedure and familiarized themselves in each chemesthetic perception by evaluating the second strongest sample (D, Table 1) from each modality, and, if not identified correctly, they evaluated the strongest sample (E, Table 1). These sample demonstrations were named and served in order (1) astringency, (2) pungency, and (3) cooling. This was done to prevent the element of surprise of the samples. After the familiarization, the participants answered the background information questionnaire while resting their senses before continuing to the sample series.

Participants evaluated three different chemesthetic modality-based sample series. These include six samples in different concentration levels with only one chemesthetic compound per series. At first, they started evaluating the cooling sample series. After that, they continued to the astringency sample series, and finally, they ended the evaluation with pungency. Samples in each series were served in two separately randomized lines. Participants started by evaluating the first three samples in the mildest concentration (zero sample, A and B; Table 1) and continued to the three strongest samples (C, D, and E; Table 1). In this study, participants focused on evaluating the intensity of given chemesthetic stimuli, not identifying it.

All samples were served in separate glass beakers marked with three-digit codes. The serving amount for every sample was 5 mL and the participants were guided to sip the entire sample, spin it around their mouth for five seconds, and then spit it out. They were also guided to wait a moment (10 s) due to the possible delay of the stimuli. Instructions also included rinsing the mouth with neutral active carbon filtered water and, if needed, eating a piece of neutral cream cracker between samples.

Participants evaluated and rated the intensity level on each chemesthetic sample once by using the line scale. The scale was anchored both verbally and numerically from 0 to 10 (0 = “no sensation”, 2 = “very mild”, 4 = “quite mild”, 6 = “quite strong”, 8 = “very strong”, and 10 = “extremely strong”). In addition, we gave both oral and written instruction descriptions to help using the line scale. The value 0 was instructed to be equal to pure water and 5 would be clearly detectable chemesthetic perception and 10 as being strong in intensity that you would want to spit out immediately. The sensory evaluation session was planned to be motivating and positive for study participants.

### 2.4. Data Analyses

After the sensory evaluation sessions, the collected data was remodeled with Microsoft Excel (Microsoft Office 2016) for further data treatment. The statistical analysis was performed with IBM SPSS Statistic 27.0 (IBM Corporation, Armonk, NY, USA). The number of participants who completed the whole series was for astringency *N* = 197 (8 excluded), pungency *N* = 199 (6 excluded), and cooling *N* = 198 (7 excluded). The zero samples (water) were excluded later from the analyses as being too mild for the test conditions due to the inconsistent evaluation by most of the participants. This was done based on previous tests and findings in the research project [14].

#### 2.4.1. Hierarchical Clustering

An agglomerative hierarchical clustering was performed with the squared Euclidean distance measure and Ward’s method on each chemesthetic modalities from the intensity results. A range of three-cluster model was selected based on the effect size of every cluster for the hierarchical clustering analyses. From these formed three cluster levels, the differences in intensity ratings were measured with a one-way multivariate analysis of variance (MANOVA) and with a series of Tukey’s post hoc tests in every sample concentration.

#### 2.4.2. Oral Chemesthesis Sensitivity Score

The data studied in this research was acquired from 196 participants for the oral chemesthesis sensitivity score (CSS) measurements. The oral chemesthesis sensitivity score was defined based on the compounds selected for this study using a similar method as the taste sensitivity score (TSS), which was measured earlier in the FoodTaste project from the same set of participants (*N* = 189) [14]. The rating for the oral chemesthesis sensitivity was created based on the average from the astringency, pungency, and cooling sensitivity groups also called clusters (1, 2, and 3). From these three chemesthetic modalities and the formed clusters using the hierarchical clustering method, it was possible to generate a total of seven sensitivity groups (1.00, 1.33, 1.67, 2.00, 2.33, 2.67, and 3.00). These sensitivity groups were classified into three sensitivity categories: Hyposensitive (CSS: 1.00 and 1.33), Semi-sensitive (CSS: 1.67, 2.00, and 2.33), and Hypersensitive (CSS: 2.67 and 3.00).

#### 2.4.3. Sensitivity Group Interactions

The interaction between each modality’s sensitivity group was studied with a multinomial logistic regression model by selecting other chemesthetic and taste modalities as explaining predictors. First, we created and explored the base for chemesthesis modality interactions using multinomial logistic regression and all the chemesthesis cluster data. Then, the base model was extended to study the interaction between chemesthesis and taste cluster data. In both phases, we used forward and backward stepwise selection techniques. The largest clusters from each modality were chosen as a reference category in every case and the criterion significance was *p* ≤ 0.05. If the odds ratio (OR) was rated less than 1.00, then the explaining factor belonged 1/OR times more likely to the reference group instead of the dependent group.

The correlation between chemesthesis and taste was measured by calculating Pearson’s correlation coefficient that included every modality. The correlation test included the sensitivity scores and modality-specific sensitivity groups of chemesthesis and taste.

The participants that evaluated both chemesthesis and taste tests (*N* = 189) were qualified for the interaction tests between chemesthesis and taste.

## 3. Results

### 3.1. Participant Characteristics

From the volunteered participants (*N* = 205) that attended the chemesthesis sensory evaluation test, the majority were women (79.5%). The average age was 41.7 ± 15.2 years with a range of 19–79 years. Both gender and age groups were selected as background variables for this study. Detailed information on the structure of gender and age groups is in Table 2.

### 3.2. Hierarchical Clustering

Three sensitivity clusters (CSG1 = a least sensitive, CSG2 = a semi-sensitive and CSG3 = a most sensitive) were formed for each chemesthetic modality based on means and standard deviations. The higher the concentration of each compound, the higher the intensity rate was. All results and sizes of clusters are shown in Table 3. Each modality is presented in separate paragraphs. In addition, a statistically significant one-way MANOVA effect was obtained with four test statistics (Pillai’s Trace; Wilk’s Λ, Hotelling’s Trace, and Roy’s Largest Root), even when some variables did not have homogeneity of variance-covariance as an assumption. These results showed that there are differences between the mean of the sample concentrations and clusters, and hence, the comparison across three cluster levels was studied with a series of post hoc analyses (Tukey’s test) in every sample concentration. 

#### 3.2.1. Astringency

Based on astringency intensity clusters, the subjects were classified into three oral chemesthesis sensitivity groups: A-CSG1 (*N* = 91), A-CSG2 (*N* = 62), and A-CSG3 (*N* = 44) and the clusters were significantly different in every concentration (*p* ≤ 0.001). The sensitivity groups of astringency are shown in Table 3.

Cluster 1 (the least sensitive, A-CSG1) consisted of 46% of the participants (91 of 197) and was the largest cluster in astringency. They rated all intensities milder than the overall mean (*N* = 197), cluster 2 or cluster 3 members. In cluster 1, the mean of the strongest sample E was 6.21 indicating it was perceivable but not strong.

Cluster 2 (the semi-sensitive, A-CSG2) was the second-largest cluster with 32% of the participants (62 of 197). Samples A and B were significantly different from cluster 3 but not with cluster 1. The samples C, D, and E were different from cluster 1 but not with cluster 3.

Cluster 3 (the most sensitive, A-CSG3) with 22% of the participants (44 of 197) had the highest intensity ratings for every sample. These participants in this group perceived astringency in low concentration samples A and B, which separated them from cluster 2. The C, D, and E samples, however, were significantly different from cluster 1 but not from cluster 2.

#### 3.2.2. Pungency

Pungency clusters had statistically significant differences in every concentration (*p* ≤ 0.001). Based on the pungency intensity clusters, the subjects were classified into three oral chemesthesis sensitivity groups: P-CSG1 (*N* = 56), P-CSG2 (*N* = 59), and P-CSG3 (*N* = 84). The sensitivity groups of pungency are shown in Table 3.

Cluster 1 (the least sensitive, P-CSG1) consisted of 28% of the participants (56 of 199) being the smallest group. They rated all intensities milder than the overall mean (*N* = 199), cluster 2 or cluster 3 members. In cluster 1, the mean of the strongest sample E was 5.18 indicating that it was perceivable but not strong.

Cluster 2 (the semi-sensitive, P-CSG2) was the second-largest cluster with 30% of the participants (59 of 199). From samples A, B, and C the samples differed significantly from cluster 3, but it was not statistically different from cluster 1. Samples D and E were different from clusters 1 and 3. In cluster 2, the mean of the strongest sample E was 8.15 indicating that it was perceivable and strong.

Cluster 3 (the most sensitive, P-CSG3) with 42% of the participants (84 of 199) was the largest group and it had the highest intensity ratings for every sample. Cluster 3 differs statistically significantly from clusters 1 and 2 in every sample. The strongest sample E was rated to 8.75 which was perceivable and strong.

#### 3.2.3. Cooling

Cooling clusters had statistically significant differences in every concentration (*p* ≤ 0.001). Based on the cooling intensity clusters, the subjects were classified three oral chemesthesis sensitivity groups: C-CSG1 (*N* = 81), C-CSG2 (*N* = 96), and C-CSG3 (*N* = 21). The sensitivity groups of cooling are shown in Table 3. All the cooling clusters (1, 2, and 3) were significantly different from each other on every concentration level.

Cluster 1 (the least sensitive, C-CSG1) consisted of 41% of the participants (81 of 198). They rated all intensities milder than the overall mean (*N* = 198), cluster 2 or cluster 3 members. In cluster 1, the mean of the strongest sample E was 3.99 that indicates it was perceivable but not strong.

Cluster 2 (the semi-sensitive, C-CSG2) was the second-largest cluster with 48% of the participants (96 of 198). This was noticed to be the largest group.

Cluster 3 (the most sensitive, C-CSG3) with 11% of the participants (21 of 198) was the smallest group and it had the highest intensity ratings for every sample. The strongest sample E was rated to 8.78 which was perceivable and strong.

### 3.3. Oral Chemesthesis Sensitivity Score

**Figure 1:**
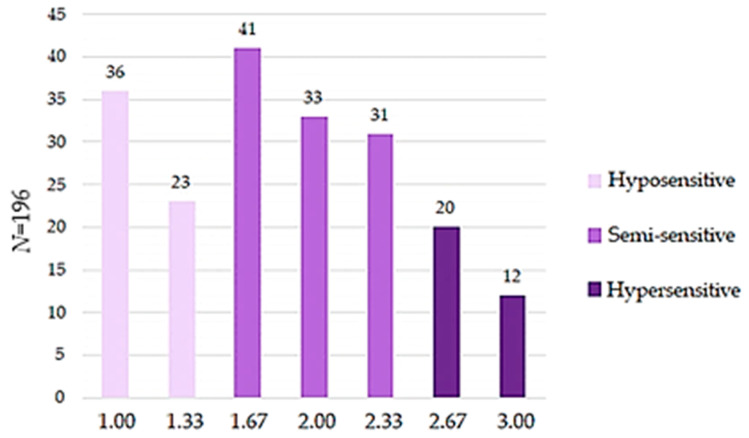
The participants were divided into different sensitivity groups (Hyposensitive *N* = 59, Semi-sensitive *N* = 105, and Hypersensitive *N* = 32), based on their oral chemesthesis sensitivity score (CSS) value presented in X-axis. The number of participants in each group is shown in the Y-axis.

Chemesthesis modality-based sensitivity groups were summed for each participant and averaged into the oral chemesthesis sensitivity score (CSS). The distribution of CSS is shown in Figure 1. For the majority of participants (53.6%, 105 of 196) CSS ranged between 1.67 and 2.33, and therefore, they were classified to the semi-sensitive group. For the hyposensitive group (30.1%, 59 of 196) CSS ranged between 1.00 and 1.33. In the hyposensitive group, 36 subjects (18.4%) belonged to the least sensitive cluster in every chemesthetic modality. The minority of participants (16.3%, 32 of 196) had CSS above 2.67 being classified to the hypersensitive group. There were only 12 participants (6.1%) who belonged to the most sensitive cluster in every chemesthetic modality.

### 3.4. Chemesthesis Sensitivity Group Interactions

The odds ratio model for the statistically significant interactions of chemesthesis sensitivity groups for each chemesthetic modality is presented in Table 4. Interactions were studied and modeled independently by the multinomial logistic regression. Each chemesthetic modality was chosen as a dependent variable and other modalities were set as explaining factors.

#### 3.4.1. Astringency

The multinomial logistic regression shows that 53.6% of the astringency sensitivity group membership was classified correctly when other chemesthesis groups were explaining factors in Table 4. Those who perceived capsaicin elicited pungency weakly (P-CSG1) were predicted to be 2.78 * times more likely in the least sensitive group (A-CSG1) than semi-sensitive (A-CSG2) in astringency. Additionally, the least sensitive group in pungency (P-CSG1) were 3.70 * times more likely to be in the least sensitive group (A-CSG1) than the most sensitive group (A-CSG3) in astringency. Participants who were classified into semi-sensitive pungency group (P-CSG2), were 4.00 * times more likely to be in the least sensitive astringency group A-CSG1 than A-CSG3. Those who perceived cooling as the least sensitive (C-CSG1) were 4.00 * times more likely to be in the least sensitive astringency group (A-CSG1) than the most sensitive group (A-CSG3). Those who were classified into the most sensitive cooling group (C-CSG3), were 6.27 ** times more likely to be in the most sensitive astringency group (A-CSG3) than the least sensitive (A-CSG1).

#### 3.4.2. Pungency

Furthermore, in the pungency sensitivity group, the estimated 57.1% of members classified correctly when other chemesthesis groups were predictors in Table 4. The subjects who perceived astringency samples on a semi-sensitive level (A-CSG2) were 2.78 * times more likely to perceive pungency most strongly (P-CSG3) than weakly (P-CSG1). Those who were most sensitive to astringency (A-CSG3) were 3.70 * times more likely to perceive pungency most strongly (P-CSG3) than weakly (P-CSG1), and 4.00 * times more likely to be most sensitive in pungency (P-CSG3) than semi-sensitive (P-CSG2). Participants who were least sensitive in cooling (C-CSG1) were 5.70 *** times more likely to perceive pungency weakly (P-CSG1) and 2.67 * times more likely to perceive pungency on semi-sensitive level (P-CSG2) than strongly (P-CSG3).

#### 3.4.3. Cooling

Finally, in cooling the estimation of 62.2% members correctly predicted when other oral chemesthesis sensitivity groups were predictors in Table 4. Those who were classified into the most sensitive astringency group (A-CSG3) were 4.00 * times more likely to be in the semi-sensitive group in cooling (C-CSG2) than the least sensitive group (C-CSG1). Additionally, the most sensitive group in astringency (A-CSG3) was 6.27 ** times likely to be in the most sensitive group in cooling (C-CSG3) than in the semi-sensitive group in cooling (C-CSG2). Participants who were the least sensitive to pungency (P-CSG1) were 5.70 *** times more likely to perceive cooling as the least sensitive (C-CSG1) than the semi-sensitive (C-CSG2). Additionally, those who perceive pungency on the semi-sensitive level (P-CSG2) were 2.67 * times more likely to be the least sensitive to cooling (C-CSG1) than the semi-sensitive (C-CSG2).

### 3.5. Interactions between Chemesthesis and Taste Modality-Specific Sensitivity Groups

**Figure 2:**
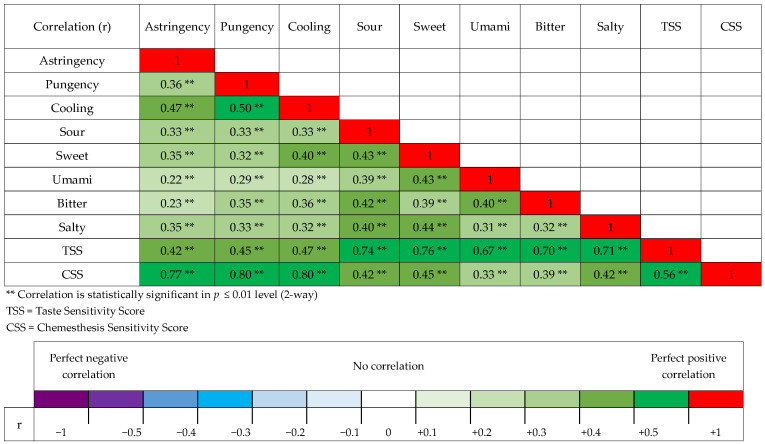
The Pearson correlation matrix between chemesthesis and taste variables *N* = 189. In the matrix, the numeric r-value and color-coding indicate the level of correlation with tested factors. The taste qualities and sensitivity score were measured earlier [14].

Pearson correlation coefficient test results between chemesthesis (astringency, pungency, cooling, and CSS) and taste (sour, sweet, umami, bitter, salty, and TSS) are presented in Figure 2. The results show a statistically significant positive correlation between all the analyzed chemesthesis and taste factors. The correlation between the oral chemesthesis and taste sensitivity scores was r = +0.56 ** and the coefficient of determination was r^2^ = 0.31 **. This indicates that the linear relationship between chemesthesis and taste scores can explain 31% of the total variation.

The odds ratio model for the statistically significant interactions of chemesthetic modalities in sensitivity explained by taste modalities using the multinomial logistic regression is presented in Table 5 which is an extension of Table 4. Interactions were studied and modeled independently by the multinomial logistic regression. The taste modality-based sensitivity groups were set as an explaining factor and the oral chemesthesis sensitivity groups as dependent factors in the multinomial logistic regression (*N* = 189). As before, the largest cluster was set as a reference category in every case. Similarly, the odds ratio model for the statistically significant interactions of taste modalities in sensitivity explained by chemesthesis modalities using multinomial logistic regression is presented in Table 6 which was the extension of Puputti et al. (2018) created model. Only the statistically significant results are reported in Table 4, Table 5 and Table 6.

#### 3.5.1. Astringency and Taste Modalities

In astringency, when all the other chemesthetic modalities were combined with taste modalities in Table 5; the correctly predicted rates for astringency were with sour = 55.0%, salty = 56.0%, sweet = 51.3%, bitter = 55.6%, and umami = 55.0%. All the taste modalities could predict astringency on some level. Those who belonged to the least sensitive group in sour taste (SO-TSG1), were 5.56 *** times more likely to be least sensitive in astringency (A-CSG1) rather than semi-sensitive (A-CSG2). The ones who were most sensitive in salty taste (SA-TSG3) were 4.72 * times more likely to belong in the most sensitive group in astringency and 3.51 * times more likely to being semi-sensitive group (A-CSG2) rather than A-CSG1. For a sweet taste, the most sensitive group (SW-TSG3) was 3.64 * times more likely to belong in the most sensitive group in astringency (A-CSG3) and 3.66 * times more likely in the semi-sensitive astringency group (A-CSG2) rather than the least sensitive (A-CSG1). In addition, those in the semi-sensitive sweet taste group were 2.23 * times more likely to be also the semi-sensitive group in astringency (A-CSG2) rather than least sensitive in astringency (A-CSG1). In bitter taste groups, those in the least sensitive group (BI-TSG1) were 4.55 * times more likely to belong to the least sensitive group in astringency (A-CSG1) than the semi-sensitive (A-CSG2). Similarly, the ones who were least sensitive in umami taste (UM-TSG1) were 3.45 * times more likely to belong the least sensitive in astringency (A-CSG1) than semi-sensitive (A-CSG2).

#### 3.5.2. Pungency and Taste Modalities

The taste modalities: salty, sweet, and bitter were able to predict pungency when they were combined with other chemesthetic modalities separately in Table 5. The correctly predicted rates for pungency were with salty = 58.7%, sweet = 59.3%, and bitter = 55.6%. Those who were semi-sensitive in salty taste were 2.78 * times more likely to belong to the most sensitive group in pungency rather than the least sensitive group. They were also 3.34 * times more likely to belong in the most sensitive (P-CSG3) than the semi-sensitive group (P-CSG2) in pungency. For sweet, the semi-sensitive group (SW-TSG2) was 2.44 * times more likely to belong in the most sensitive group in pungency (P-CSG3) than semi-sensitive (P-CSG2). Finally, the most sensitive bitter tasters (BI-TSG3) were 3.23 * times more likely to belong to most sensitive in pungency (P-CSG3) than least sensitive (P-CSG1). Interestingly, those who were least sensitive in bitter taste (BI-TSG1), were 5.26 * times more likely to belong in the most sensitive in pungency (P-CSG3) than the semi-sensitive group (P-CSG2).

#### 3.5.3. Cooling and Taste Modalities

The cooling could be predicted by sour, sweet, and bitter taste sensitivity when combined with other chemesthetic modalities separately in Table 5. The correctly predicted rates for cooling were with sour = 68.3%, sweet = 63.5%, and bitter = 63.5%. The most sensitive sour tasters (SO-TSG3) were 6.15 ** times more likely to belong in the most sensitive group in cooling (C-CSG3) than semi-sensitive sensitive (C-CSG2). For sweet tasters, the most sensitive were 4.00 ** times more likely to belong in the semi-sensitive group in cooling (C-CSG2) than least sensitive (C-CSG1). Similarly, the semi-sensitive sweet tasters were 2.63 * times more likely to be semi-sensitive (C-CSG2). The most sensitive bitter tasters (BI-TSG3) belonged 2.38 * times more likely to the semi-sensitive group in cooling than least sensitive C-CSG1.

#### 3.5.4. Sour Taste and Chemesthetic Modalities

When sour taste was set up as a dependent factor in Table 6; the statistically significant chemesthetic explaining factors were discovered in astringency with the correctly predicted rate of 64.0%, and cooling at 67.2%. If individuals were classified into the semi-sensitive group in astringency (A-CSG2), they were 5.26 ** times more likely to belong the semi-sensitive group (SO-TSG2) than the least sensitive group (SO-TSG1) in sour taste. Moreover, the most sensitive individuals in cooling (C-CSG3) were 5.01 ** times more likely belong to the most sensitive group (SO-TSG3) than the semi-sensitive (SO-TSG2) in sour taste.

#### 3.5.5. Sweet Taste and Chemesthetic Modalities

In the case of sweet taste as a dependent factor in Table 6; the statistically significant chemesthetic explaining factors were discovered in all the chemesthetic modalities. The correctly predicted ratings were in astringency = 58.2%, pungency = 61.4%, and cooling = 58.7%. If individuals were classified into the most sensitive group in astringency (A-CSG3), they were 4.45 * times more likely to belong the most sensitive group (SW-TSG3) than the least sensitive group (SW-TSG1) in sweet taste.

Moreover, individuals who belonged to the semi-sensitive group in pungency (P-CSG2) were 2.94 * times more likely to belong the least sensitive group (SW-TSG1) than the semi-sensitive (SW-TSG2) in sweet taste.

Finally, those who perceived cooling as the least sensitive (C-CSG1), were 2.50 * times more likely to belong into the least sensitive group (SW-TSG1) than the semi-sensitive group (SW-TSG2) in sweet taste.

#### 3.5.6. Salty Taste and Chemesthetic Modalities

The salty taste could be explained with astringency and pungency in Table 6. The correctly predicted values were in astringency 65.6% and pungency 61.9%. The most sensitive individuals in astringency (A-CSG3) were 4.98 ** times more likely to belong into the most sensitive group (SA-TSG3) than the least sensitive group (SA-TSG1) in salty taste. Furthermore, those who were semi-sensitive in pungency (P-CSG2) were 4.00 * times more likely to belong into the least sensitive group (SA-TSG1) than the most sensitive group (SA-TSG3) in salty taste.

#### 3.5.7. Bitter Taste and Chemesthetic Modalities

When bitter taste was set as the dependent factor in Table 6, the statistically significant explaining chemesthetic factors were found in pungency with 61.4% correctly prediction rate and cooling with 58.2%. Individuals in the least sensitive group in pungency (P-CSG1) were 2.86 * times more likely to belong the semi-sensitive group (BI-TSG2) than the most sensitive group (BI-TSG3) in a bitter taste. Similarly, individuals who belonged to the least sensitive group in cooling (C-CSG1) were 2.22 * times more likely to belong into the semi-sensitive group (BI-TSG2) than the most sensitive group (BI-TSG3) in bitter.

#### 3.5.8. Umami Taste and Chemesthetic Modalities

According to our analysis, the oral chemesthesis sensitivity groups could not statistically significantly explain the sensitivity groups in umami taste.

## 4. Discussion

### 4.1. Chemesthesis Sensitivity Segmenting with Hierarchical Clustering

This study applied hierarchical clustering to segment individuals into different oral chemesthesis sensitivity groups for the first time based on our knowledge. The hierarchical clustering method has been proven to be a valid tool that can take into account all the samples based on taste sensitivity research [14]. The method has been used before in chemesthesis-related studies that focus on the association between taste and chemesthesis [12,31,32]. Beyond chemosensory science, hierarchical clustering has been the more common statistical approach to consumer segmentation in marketing and consumption research [33,34,35,36].

All the chemesthetic modalities formed three clusters that were perceived on different sensitivity levels according to the one-way MANOVA in Table 3. These clusters were classified into different sensitivity groups. In the case of astringency perception, none of the clusters can be labeled easily as the semi-sensitive group. However, a clear difference between the least sensitive (A-CSG1) and the most sensitive (A-CSG3) groups was detected on each aluminum sulfate concentration. Next, in pungency perception, the three different sensitivity groups were detected in high concentration capsaicin samples (D and E). In lower concentrations (A, B, and C) the least sensitive (P-CSG1) and the most sensitive (P-CSG3) groups were different. Finally, in cooling perception, all three detected sensitivity groups were statistically significantly different on each menthol concentration level.

Our results show that similarly to taste [14]; the individuals can be classified into different sensitivity groups in the perception of chemesthesis. Moreover, analyzing oral chemesthesis sensitivity with several different concentration levels made it easier to determine the type of sensitivity groups.

### 4.2. Oral Chemesthesis Sensitivity

Results show that we were able to illustrate the oral chemesthesis sensitivity with special CSS developed in this study. Most participants (53.6%) were classified into the semi-sensitive group and the rest (46.4%) to the extreme groups (hyposensitive and hypersensitive). Due to the different mean groups, our findings also suggest that the semi-sensitive group is more heterogeneous than the extreme groups based on the oral chemesthesis sensitivity score. There was a more possible combination of means for the semi-sensitive groups than in hyposensitive and hypersensitive groups. If the participants were extremely sensitive to pungency and cooling but not sensitive to astringency, then they were classified into the semi-sensitive group. If they were extremely sensitive to pungency but not for cooling and astringency, then they were classified into the semi-sensitive group also. This proves that although there was a positive correlation between chemesthetic modalities, there can also be different subgroups who might have a higher or lower threshold to certain chemesthetic irritants.

This study focused on measuring the oral chemesthesis sensitivity, and then, the correlation between selected modalities of chemesthesis and taste. It is possible that personal background factors explaining taste sensitivity (gender, age, BMI, and smoking status) [37] could also explain sensitivity to chemesthesis. In addition, the composition of saliva is likely to interact with chemesthesis receptors since saliva controls flavor release [38]. Therefore, it is possible that saliva has a role in explaining individual oral chemesthesis sensitivity. Moreover, the Italian Taste project has discovered that genetic factors such as *hTAS2R38* can influence the intensity of astringency of aluminum sulfate [39]. This is interesting since studies have stated that the intensity of astringency sensation can be associated to PROP (6-n-propylthiouracil) bitter taste [6,21] in wines. Furthermore, genetic variation in the TAS2R38 taste receptor has been studied to influence the consumption of lingonberries [6], and thus, indicating that chemesthesis can have a role in our food choices.

### 4.3. Oral Chemesthesis Sensitivity Group Interactions

We found that chemesthesis clusters were linked together based on the predicted connections between every chemesthetic modality (Table 4). All the chemesthetic modalities had statistically significant interactions with each other. The strongest interactions of 6.27 ** were discovered between the most sensitive clusters in cooling (C-CSG3) and astringency (A-CSG3). Moreover, the least sensitive groups in pungency (P-CSG1) and cooling (C-CSG1) had also a relatively strong interaction of 5.70 *** that was highly significant. All chemesthetic modalities seem to have interactions between one another according to the prediction rates. These results differ from the earlier measured taste interaction model. For example, Puputti et al. (2018) found out that insensitivity to umami predicted intensity to bitter but bitter insensitivity did not predict umami insensitivity [14]. These findings prove that chemesthesis sensitivity has more interactions between its modalities than with taste modalities. Similar chemesthetic interaction was found between pungency (Sichuan pepper oleoresin) and heat (capsaicin) in research that used a similar methodology of measuring and analyzing individual sensitivity [40].

### 4.4. Interactions between Chemesthesis and Taste Sensitivity

Based on the sensitivity tests, both the CSS and the TSS had the majority of participants classified into the semi-sensitive groups. The correlation matrix showed positive and statistically significant correlations with each tested chemesthetic and taste modalities. The highest correlations were found between the CSS and chemesthetic modalities (astringency, pungency, and cooling), and between TSS and taste modalities (sweet, sour, salty, bitter and umami). Results show that there was also a positive and statistically significant correlation between the oral chemesthesis sensitivity and taste sensitivity (r = +0.56 **, Figure 2). The positive correlation indicates that if individuals are sensitive in taste perception, they are more likely to be sensitive to chemesthesis. This indicates that specific taste and chemesthesis sensitivities might not exclude the sensitivity to one or another. However, our results also state that the linear relationship between the chemesthesis and taste scores can only explain 31% of the total variation. Other studies state that subjects that are highly responsive to taste modalities are also highly responsive to the astringency and pungency of capsaicin [32].

Sensitivity groups in chemesthetic modalities can be statistically significantly explained by taste modalities (Table 5). Astringency can be explained by all the taste modalities: sour, sweet, salty, bitter, and umami. Unlike astringency, pungency, and cooling could only be predicted by some of the tested taste modalities. The strongest taste modality-based interaction (6.15 **) was noticed between the most sensitive group in cooling (C-CSG3) and the most sensitive in sour taste (SO-TSG3). The least sensitive groups in astringency (A-CSG1) and sour taste (SO-TSG1) had the interaction (5.56 ***) that was strong and highly significant. These results indicate that astringency has more dimensions to taste sensitivity when compared with other chemesthetic modalities. Furthermore, results suggest that strong associations between chemesthesis and taste are dependent on the modalities.

The oral chemesthesis sensitivity modalities can explain the taste sensitivity groups shown in Table 6. We noticed that sweet taste was the only modality that could be explained by all the chemesthetic modalities. The semi-sensitive group in astringency (A-CSG2) had the strongest and statistically significant interaction (5.26 **) with the semi-sensitive group in sour taste (SO-TSG2). Moreover, the most sensitive group in cooling sensitivity (C-CSG3) was noticed to strongly interact with the most sensitive group in sour taste (5.01 **) (SO-TSG3). The most sensitive group in astringency (A-CSG3) was found to be in strong interaction (4.98 **) with the most sensitive group in salty taste (SA-TSG3).

We found out that relatively direct and statistically significant interactions between chemesthesis and taste modalities (Table 5 and Table 6) were found between the most sensitive groups in astringency (A-CSG3) and sweet taste (SW-TSG3) with OR of 4.45 * and 3.64 *. In addition, the most sensitive groups in astringency (A-CSG3) and salty taste (SA-TSG3) had similar statistically significant interaction with OR of 4.98 ** and 4.72 *. The pungency sensitivity was a significant predictor for semi-sensitivity in saltiness and sweetness based on the multinomial logistic regression model (Table 5). Moreover, insensitivity in sweetness and saltiness were significant predictors for semi-sensitivity in pungency. The interaction between sensitivity in cooling and sourness was significant (Table 5 and Table 6). Although astringency was predicted by all the taste modalities, it did not predict statistically significantly bitter and umami taste.

Surprisingly, our results showed that different oral chemesthesis sensitivity groups could not predict the sensitivity to umami. The reason for this might be linked to the genetic variations of TRP channels and their potential interaction with the gustatory system [31]. Taste perception studies with trigeminal stimuli of capsaicin have shown that the presence of capsaicin in the peri-threshold range reduced taste thresholds for sweet, sour, salty, and bitter but not for umami [41]. Also, a previous study focusing on chemical heat has shown that sweet, sour, salty, and bitter are influenced by the pungency of capsaicin, however, capsaicin did not influence umami [31,42]. Moreover, this supports our positive correlation findings by suggesting that the oral chemesthesis increases taste sensitivity. In the case of menthol and sugar [30], the increase of cough reflex and our findings positive correlation could indicate that there might be an interaction between cooling and sweet.

The oral chemesthesis has been reported to use primarily the *Trigeminal* (V) nerve, and secondarily the *Glossopharyngeal* (IX) and the *Vagus* (X) nerves as a neural pathway [10]. Furthermore, the *Glossopharyngeal* (IX) and the *Vagus* (X) are also associated with taste perception [43]. Therefore, the correlation with taste might be explained partly by the neural activity of the *Glossopharyngeal* (IX) and the *Vagus* (X) nerves. In contrast, we can assume that the correlation with taste would have been higher if taste modalities used the *Trigeminal* (V) nerve as a primary neural pathway. We can see from the correlation matrix (Figure 2) that taste modalities have a higher correlation with each other than oral chemesthesis. Interoperation would be that if the modalities are using the same neural pathway primarily, then the correlation would be on a similar level with other modalities that use the same primary pathway.

In addition, Robino et al. (2022) have shown [39] that PROP taster status was associated with taste (sourness of citric acid, saltiness of sodium chloride, bitterness of caffeine, savory (umami) of monosodium glutamate, and sweetness of sucrose) and chemesthesis (astringency of aluminum sulfate, and pungency of capsaicin) intensity. Their results also suggest that the *hTAS2R38* genotype is the most important variable for explaining sensitivity differences in astringency and taste. Moreover, gender was noted to be the primary determinant for pungency sensitivity. These results indicate that the *hTAS2R38* genotype and gender may also explain the individual differences in our study. For further studies, the association between PROP taster status and chemesthesis sensitivity will also be suspected. Zhang et al. (2021) studied the correlation between taste and chemesthesis by applying pungency (Sichuan pepper oleoresin), heat (capsaicin), bitterness (PROP), and saltiness (NaCl) [40]. Their study showed that the sensitivity to bitterness could be partly predicted by the heat of capsaicin and pungency of Sichuan pepper oleoresin.

The oral responsiveness to taste, pungency, and astringency sensations are linked to the consumption [18] and preferences [17] of alcoholic beverages. Therefore, individual differences in the oral chemesthesis sensitivity may be associated with the occurrence of alcohol-related health risks. Furthermore, the individual profiles in the oral chemesthesis and taste sensitivity could explain certain illnesses such as the pathophysiology of primary burning mouth syndrome (BMS) [44]. Reflected to that, we suggest that studying the association between oral chemesthesis sensitivity using our methods, and saliva structure could give more detailed information on the possible critical role of gustatory and somatosensory profiles in BMS.

### 4.5. Strengths and Limitations

These results show that it is possible to measure the oral chemesthesis sensitivity with CSS. To our knowledge, this seems to be the first time when the generalized oral chemesthesis sensitivity has been measured from human beings by using a series of different chemesthetic modalities (astringency, pungency, and cooling) with different concentration levels. We were able to create a model that can demonstrate individual sensitivity on different levels.

This study measured individual chemesthetic perception with untrained sensory study participants, so there is a possibility of scale-use bias. The participants did not use references samples for evaluating the intensity with the line scale. On the other hand, they were given written and oral instructions on how to use the scale. Only one prototypic oral chemesthetic sample was used to test each modality. Alternative chemesthetic substances may use different channels for transduction. Therefore, individuals may be more or less sensitive to other prototypic samples in astringency, pungency, or cooling.

This study focused on measuring the oral chemesthesis sensitivity. If we want to measure overall chemesthesis sensitivity, we need to notice other chemesthetic modalities such as nasal chemesthesis. Studies have shown that blocking nasal pathways may decrease the chemesthetic perception of foods [39]. In addition, Haley and McDonald (2016) highlight that menthol compounds have strong minty aromas and flavors as well as a cooling effect [7]. Therefore, the nasal chemesthetic or odor perception might affect results since we did not block the nasal airways. The samples were planned to be neutral in aroma and blocking of participants’ nasal pathways might cause discomfort.

A study on the oral cavity mucosae and regional sensitivity suggests that differences in sensitivity and the extent of desensitization among areas of the mouth (tongue, cheek, hard palate, and lip) are responding but impacted differently to capsaicin [45]. Therefore, liquid samples were preferred in this study to reach each area in terms of measuring the oral chemesthesis sensitivity. Furthermore, their study also discovered a delay in reaching a maximum intensity of capsaicin in the hard palate. This proves that when measuring the sensitivity to chemesthetic compounds, the delay can influence results if samples are evaluated too quickly without a resting period and rinses between samples. Nevertheless, these issues were noticed in our sensory study, and we trusted that participants followed the given instructions.

Moreover, studies have shown that using different oils instead of water in samples can create a higher threshold to capsaicin elicited pungency perception [46]. Therefore, in the food-related concept, it needs to be noticed that there are factors that can reduce the sensitivity rate in chemesthesis. Studies focusing on mixed solutions between chemesthetic, and taste modality are needed to understand the cross-modality of chemesthetic perception better.

Our research results did not include analysis of other chemesthetic modalities such as metallic, pungency of carbonation, pain, or fatty perceptions. However, measuring every chemesthetic modality and stimulating irritants takes a lot of time and effort from participants. In addition, ethical aspects need to be followed since chemesthetic stimuli can activate pain perception, which is why it is important to plan sensory studies carefully, noticing the individual differences.

The concentration level of the samples was tested experimentally due to the lack of standardized methods to measure the oral chemesthesis sensitivity. Too low or high concentrations might cause bias, and thus, make detecting different sensitivity groups challenging. Nevertheless, we found out that there were significant differences, and we were able to find three different sensitivity groups from each chemesthetic modality.

Background factors may affect the results of this study. However, we already know based on the test setup, that gender and age distribution were unbalanced. The main aim of the study was to test whether it is possible to measure individual oral chemesthesis sensitivity from the substantial number of participants, which was successfully executed.

## 5. Conclusions

Our findings give a better understanding of the fundamental role of the oral chemesthesis sensitivity to flavor experiences of foods. The study shows that the individual sensitivity in oral chemesthetic perception varies. Participants were classified into different sensitivity groups based on their chemesthetic modality-based intensity ratings. The oral chemesthesis sensitivity score can ideally demonstrate the individual’s generalized sensitivity to oral chemesthetic perception. However, more screening of different chemesthetic modalities and irritants is needed.

This study fills the gap in knowledge related to the interaction between the oral chemesthesis sensitivity and the taste sensitivity. The correlation between chemesthesis and taste sensitivity was positive, and also, the interactions were discovered. Other chemesthesis and taste modalities can partly predict individual modality-specific sensitivity groups in oral chemesthesis.

The individual sensitivity measurements in chemesthesis were successfully executed and in addition with taste measurements. These findings can create the basic ground for further studies focusing on food-related behavior and health.

## Figures and Tables

**Table 1:** The sample series for chemesthetic modalities was created by using activating chemical compounds. Samples are listed from the lowest concentration to the highest (A–E). Every sample was evaluated in the same session and samples were randomized.

Chemesthetic Modality	Prototypic Compound	Sample A	Sample B	Sample C	Sample D	Sample E
Astringency	^a^ Aluminum ammonium sulfate AlNH_4_(SO_4_)_2_⋅12H_2_O	0.22 mM	0.39 mM	0.70 mM	1.24 mM	2.21 mM
Pungency	^b^ Capsaicin C_18_H_27_NO_3_	0.049 µM	0.088 µM	0.154 µM	0.275 µM	0.491 µM
Cooling	^c^ Menthol C_10_H_20_O	0.013 mM	0.023 mM	0.040 mM	0.072 mM	0.128 mM

^a^ Aluminum ammonium sulfate (7784-26-1), ≥99.0%, Sigma-Aldrich (St. Louis, Missouri, USA). ^b^ Capsaicin (404-86-4), ≥98.5%, Fluka Sigma-Aldrich (St. Louis, Missouri, USA). ^c^ Menthol (2216-51-5), ≥99.7%, Symrise (Holzminden, Germany).

**Table 2:** The subject’s demographical information and classification from those who attended to evaluate all chemesthetic samples (*N* = 205).

Chemesthesis Test		*N*	%
Gender	Male	40	19.5
	Female	163	79.5
	Prefer not to disclose	2	1.0
	Total	205	100
Age group (years)	19–29	54	26.3
	30–39	51	24.9
	40–49	42	20.5
	50–59	25	12.2
	60–69	20	9.8
	70–79	13	6.3
	Total	205	100

**Table 3:** One-way MANOVA-test results between clusters and chemesthetic sample series **mean** ± standard deviations (95% confidence intervals for the mean intensities in the brackets) for every sample (A–E, Table 1), and the distribution of subjects between the oral chemesthesis sensitivity clusters (CSG1, CSG2, and CSG3).

Chemesthetic Modality	Test Statistics ^1^	Sample	CSG1 = Cluster 1	CSG2 = Cluster 2	CSG3 = Cluster 3	All
Astringency	*p* ≤ 0.001 F_df(10.0)_ = 58.7 Wilks’ Λ = 0.153 partial η^2^ = 0.608	A	**1.05** ± 1.02 (0.84–1.27) a	**0.86** ± 0.95 (0.62–1.10) a	**3.58** ± 2.25 (2.89–4.26) b	**1.56** ± 1.75 (1.31–1.81)
		B	**1.08** ± 1.11 (0.85–1.31) a	**1.18** ± 1.11 (0.90–1.47) a	**4.71** ± 2.15 (4.06–5.36) b	**1.93** ± 2.06 (1.64–2.22)
		C	**1.78** ± 1.41 (1.48–2.07) a	**4.47** ± 1.33 (4.12–4.81) b	**4.83** ± 2.57 (4.05–5.61) b	**3.30** ± 2.23 (2.99–3.61)
		D	**4.03** ± 1.72 (3.68–4.39) a	**7.00** ± 1.55 (6.60–7.40) b	**7.19** ± 1.55 (6.72–7.66) b	**5.66** ± 2.23 (5.35–5.98)
		E	**6.21** ± 1.81 (5.83–6.59) a	**8.63** ± 1.10 (8.35–8.91) b	**8.18** ± 1.40 (7.75–8.60) b	**7.41** ± 1.89 (7.14–7.67)
		n (%)	91 (46)	62 (32)	44 (22)	197 (100)
Pungency	*p* ≤ 0.001 F_df(10.0)_ = 55.1 Wilks’ Λ = 0.166 partial η^2^ = 0.593	A	**1.04** ± 1.05 (0.76–1.32) a	**0.84** ± 0.76 (0.64–1.04) a	**2.50** ± 1.94 (2.08–2.92) b	**1.60** ± 1.63 (1.37–1.83)
		B	**1.21** ± 1.08 (0.92–1.50) a	**1.03** ± 1.01 (0.76–1.30) a	**3.46** ± 1.86 (3.05–3.86) b	**2.11** ± 1.85 (1.85–2.37)
		C	**2.01** ± 1.51 (1.61–2.42) a	**1.97** ± 1.28 (1.63–2.31) a	**5.06** ± 1.70 (4.69–5.43) b	**3.29** ± 2.15 (2.99–3.59)
		D	**2.97** ± 1.61 (2.54–3.40) a	**4.98** ± 1.71 (4.52–5.43) b	**7.09** ± 1.72 (6.71–7.46) c	**5.30** ± 2.40 (4.96–5.64)
		E	**5.18** ± 1.57 (4.76–5.60) a	**8.15** ± 0.82 (7.94–8.37) b	**8.75** ± 1.10 (8.51–8.99) c	**7.56** ± 1.93 (7.28–7.83)
		n (%)	56 (28)	59 (30)	84 (42)	199 (100)
Cooling	*p* ≤ 0.001 F_df(10.0)_ = 41.0 Wilks’ Λ = 0.230 partial η^2^ = 0.521	A	**0.67** ± 0.97 (0.46–0.11) a	**1.96** ± 1.38 (1.68–2.24) b	**3.76** ± 1.51 (3.07–4.45) c	**1.63** ± 1.57 (1.41–1.85)
		B	**1.47** ± 1.12 (1.22–1.71) a	**3.01** ± 1.73 (2.67–3.34) b	**4.70** ± 1.94 (3.81–5.58) c	**2.55** ± 1.82 (2.30–2.81)
		C	**1.75** ± 1.13 (1.50–2.00) a	**3.92** ± 1.54 (3.60–4.23) b	**6.54** ± 1.61 (5.81–7.28) c	**3.31** ± 2.06 (3.02–3.60)
		D	**2.59** ± 1.59 (2.24–2.95) a	**5.06** ± 1.44 (4.77–5.40) b	**8.06** ± 1.69 (7.29–8.83) c	**4.37** ± 2.30 (4.05–4.70)
		E	**3.99** ± 1.66 (3.63–4.36) a	**6.32** ± 1.47 (6.02–6.62) b	**8.78** ± 0.97 (8.34–9.22) c	**5.63** ± 2.16 (5.33–5.93)
		n (%)	81 (41)	96 (48)	21 (11)	198 (100)

Different lower cases indicate statistically significant (*p* ≤ 0.05) differences between the clusters in a sample. ^1^ One-way MANOVA for the differences in cluster intensities. CSG = the oral chemesthesis sensitivity group.

**Table 4:** The statistically significant odds ratios (OR) for chemesthetic interactions by the multinomial logistic regression model, *N* = 196.

Modality	Reference	Dependent Factor	Explaining Factor	OR (95% Confidence Intervals)	Correctly Predicted (%)
Astringency	A-CSG1	A-CSG2	P-CSG1	0.36 * (0.15–0.87)	53.6
		A-CSG3	P-CSG1	0.27 * (0.09–0.82)	
		A-CSG3	P-CSG2	0.25 * (0.08–0.74)	
		A-CSG3	C-CSG1	0.25 * (0.08–0.76)	
		A-CSG3	C-CSG3	6.27 ** (1.58–24.88)	
Pungency	P-CSG3	P-CSG1	A-CSG2	0.36 * (0.15–0.87)	57.1
		P-CSG1	A-CSG3	0.27 * (0.09–0.82)	
		P-CSG1	C-CSG1	5.70 *** (2.48–13.13)	
		P-CSG2	A-CSG3	0.25 * (0.08–0.74)	
		P-CSG2	C-CSG1	2.67 * (1.21–5.91)	
Cooling	C-CSG2	C-CSG1	A-CSG3	0.25 * (0.08–0.76)	62.2
		C-CSG1	P-CSG1	5.70 *** (2.28–13.13)	
		C-CSG1	P-CSG2	2.67 * (1.21–5.91)	
		C-CSG3	A-CSG3	6.27 ** (1.58–24.88)	

Codes: A (Astringency), P (Pungency), C (Cooling), and CSG (Chemesthesis Sensitivity Group). * *p* ≤ 0.05, ** *p* ≤. 0.01, and *** *p* ≤ 0.001.

**Table 5:** The statistically significant odds ratios (OR) for chemesthetic modalities in sensitivity explained by taste modalities by the multinomial logistic regression (*N* = 189). The explaining taste modalities were analyzed separately to keep the reliability of the model.

Modality	Reference	Dependent Factor	Explaining Factor	OR (95% Confidence Intervals)	Correctly Predicted (%)
Astringency	A-CSG1	A-CSG2	SO-TSG1	0.18 *** (0.06–0.51)	55.0
		A-CSG3	SA-TSG3	4.72 * (1.42–15.66)	56.0
		A-CSG2	SA-TSG3	3.51 * (1.15–10.70)	
		A-CSG3	SW-TSG3	3.64 * (1.03–12.88)	51.3
		A-CSG2	SW-TSG3	3.66 * (1.28–10.51)	
		A-CSG2	SW-TSG2	2.23 * (1.01–4.94)	
		A-CSG2	BI-TSG1	0.22 * (0.07–0.75)	55.6
		A-CSG2	UM-TSG1	0.29 * (0.09–0.94)	55.0
Pungency	P-CSG3	P-CSG1	SA-TSG2	0.36 * (0.13–0.97)	58.7
		P-CSG2	SA-TSG2	0.31 * (0.10–0.97)	
		P-CSG2	SW-TSG2	0.41 * (0.17–0.99)	59.3
		P-CSG1	BI-TSG3	0.31 * (0.12–0.80)	55.6
		P-CSG2	BI-TSG1	0.19 * (0.04–0.85)	
Cooling	C-CSG2	C-CSG3	SO-TSG3	6.15 ** (1.78–21.33)	68.3
		C-CSG1	SW-TSG3	0.25 ** (0.09–0.71)	63.5
		C-CSG1	SW-TSG2	0.38 * (0.18–0.82)	
		C-CSG1	BI-TSG3	0.42 * (0.19–0.91)	63.5

Codes: A (Astringency), P (Pungency), C (Cooling), CSG (Chemesthesis Sensitivity Group), SO (Sour), SW (Sweet), UM (Umami), BI (Bitter), SA (Salty) and TSG (Taste Sensitivity Group). * *p* ≤ 0.05; ** *p* ≤.0.01 and *** *p* ≤ 0.001.

**Table 6:** The statistically significant odds ratios (OR) for taste modality-specific sensitivity groups explained by chemesthetic modalities by the multinomial logistic regression (*N* = 189). The explaining chemesthetic modalities were analyzed separately to keep the reliability of the model.

Modality	Reference	Dependent Factor	Explaining Factor	OR (95% Confidence Intervals)	Correctly Predicted (%)
Sour	SO-TSG2	SO-TSG1	A-CSG2	0.19 ** (0.06–0.62)	64.0
		SO-TSG3	C-CSG3	5.01 ** (1.52–16.52)	67.2
Sweet	SW-TSG1	SW-TSG2	P-CSG2	0.34 * (0.13–0.88)	61.4
		SW-TSG2	C-CSG1	0.40 * (0.17–0.91)	58.7
		SW-TSG3	A-CSG3	4.45 * (1.14–18.06)	58.2
Salty	SA-TSG1	SA-TSG3	A-CSG3	4.98 ** (1.47–16.91)	65.6
		SA-TSG3	P-CSG2	0.24 * (0.07–0.77)	61.9
Bitter	BI-TSG2	BI-TSG3	P-CSG1	0.35 * (0.13–0.94)	61.4
		BI-TSG3	C-CSG1	0.45 * (0.20–0.99)	58.2

Codes: A (Astringency), P (Pungency), C (Cooling), CSG (Chemesthesis Sensitivity Group), SO (Sour), SW (Sweet), BI (Bitter), SA (Salty), and TSG (Taste Sensitivity Group). * *p* ≤ 0.05 and ** *p* ≤ 0.01.

## Data Availability

Data is available on reasonable request directed at the corresponding author.
